# Supplementary material for: Genome-wide analysis highlights contribution of immune system pathways to the genetic architecture of asthma
Source: Nat Commun. 2020 Apr 15;11:1776. doi: 10.1038/s41467-020-15649-3 (PMC7160128; doi:10.1038/s41467-020-15649-3)
Supplement: Supplementary file 1 — Supplementary Information [file 41467_2020_15649_MOESM1_ESM.pdf]

Supplementary Information

Genome-wide Analysis Highlights Contribution of Immune System Pathways to the Genetic Architecture of Asthma

Han et al.

Address correspondence and reprint requests to:

Hooman Allayee, PhD
Department of Preventive Medicine
Keck School of Medicine of USC 2250
Alcazar Street, CSC202
Los Angeles, CA 90033 Phone:
(323) 442-1736
Fax: (332) 442-2764
Email: hallayee@usc.edu

Supplementary Table 1. Description of datasets used for GWAS meta-analysis.

Cohort	N	Cases/Controls	Definition of Asthma Phenotype	SNPs Included (Imputation Platform(s))
**The UK Biobank**	393,859	64,538/329,321	1) Doctor-diagnosed asthma; this field is a summary of the distinct main diagnoses codes a participant has had recorded across all his/her hospital episodes.<br>2) International Classification of Diseases version-10 (ICD10) main and secondary.<br>3) Self-reported asthma.	9,572,556 (Haplotype Reference Consortium, UK10K, and 1000 Genomes Project)
**The TAGC**	142,486	23,948/118,538	Doctor-diagnosed asthma and/or standardized questionnaires.	9,415,011 (1000 Genomes Project)<br>2,001,281 (Hapmap)

Supplementary Table 2. Secondary independent association signals at 10 of the previously unknown loci identified for asthma in the meta-analysis with the UK Biobank and TAGC.

	Lead SNP				Secondary independent lead SNP(s)							
**Locus**	**CHR**	**SNP**	**BP**	**P**	**SNP**	**BP**	**EA**	**NEA**	**EAF**	**P**	**Distance from lead SNP**	**r^2 with lead SNP**
1	3q26.2	rs17485347	169,127,519	2.5E-09	rs12107696	168,764,243	C	T	0.23	6.3E-09	-363,276	0.0107
2	5q33.3	rs11746314[a,b]	156,752,957	2.1E-12	rs4579242	156,939,498	G	T	0.35	2.3E-11	186,541	0.0830
3	6p22.2	rs766406[a]	26,319,588	5.8E-14	rs198806	26,133,616	A	G	0.41	2.6E-08	-185,972	0.0903
3	6p22.2	rs766406[a]	26,319,588	5.8E-14	rs13220495	26,441,640	T	C	0.10	3.0E-13	122,052	0.0328
4	7p22.2	rs73033536[a]	3,149,883	6.2E-13	rs111246112	3,055,099	A	T	0.94	3.3E-08	-94,784	0.0005
4	7p22.2	rs73033536[a]	3,149,883	6.2E-13	rs6952068	3,114,461	A	C	0.25	2.3E-11	-35,422	0.0239
5	9q22.33	rs1537504[a,b]	101,829,542	1.3E-11	rs41283642	101,915,887	C	T	0.97	1.3E-10	86,345	0.0027
6	9q32	rs4978607	117,508,437	4.5E-08	rs2225330	117,836,400	G	A	0.79	4.8E-08	327,963	0.0008
7	11p11.2	rs714417	45,247,176	9.5E-12	rs10838465	45,426,141	A	C	0.70	2.6E-08	178,965	0.0223
8	12q22	rs12303699[a,b]	94,582,336	7.5E-12	rs2361359	94,562,875	G	C	0.86	3.0E-09	-19,461	0.0713
9	15q25.2	rs11259930[a]	84,577,350	1.4E-12	rs144362840	84,323,095	G	GT	0.93	1.2E-08	-254,255	0.0226
10	19p13.11	rs34006614	16,442,782	1.0E-08	rs8109466	17,207,011	G	T	0.29	2.4E-08	764,229	0.0003

Identified by [a]Johansson et al. (PMID:31361310) or [b]Olafsdottir et al. (PMID: 31959851) while this manuscript was under consideration.

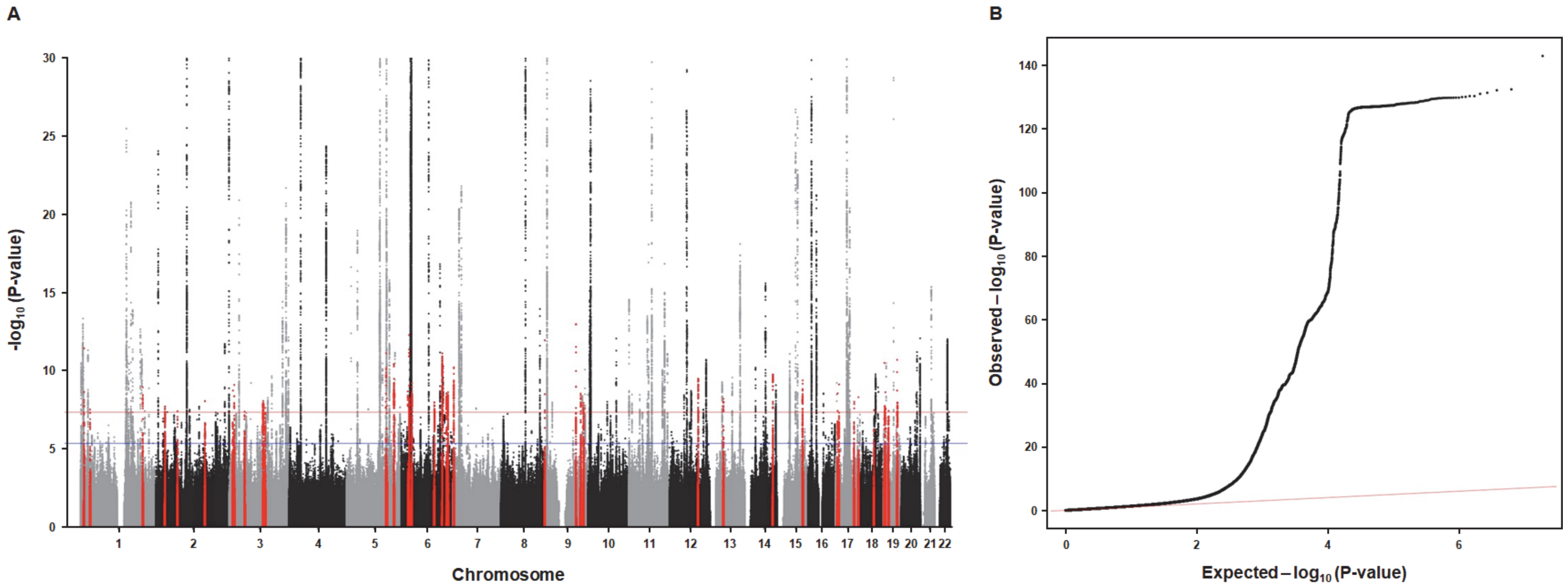

Supplementary Figure 1. Results of GWAS analysis for asthma in the UK Biobank. (A) A Manhattan plot shows 145 loci significantly associated with asthma in the UK Biobank, of which 41 loci were previously unknown (red dots). The GWAS analysis included 64,538 cases and 329,321 controls. Genome-wide thresholds for significant ($P=5.0x10^{-8}$) and suggestive ($P=5.0x10^{-6}$) association are indicated by the horizontal red and dark blue lines, respectively. P-values are truncated at $-log_{10}(P)=30$. **(B)** A quantile-quantile plot shows the observed versus the expected P-values from the association analyses for asthma in the UK Biobank. The genomic control factor (λ) in the UK Biobank GWAS results was 1.31 and the LD Score intercept from BOLT-LMM was 1.076 (SE=0.009), suggesting that any inflation of test statistics was more likely due to many small genetic effects rather than population structure.

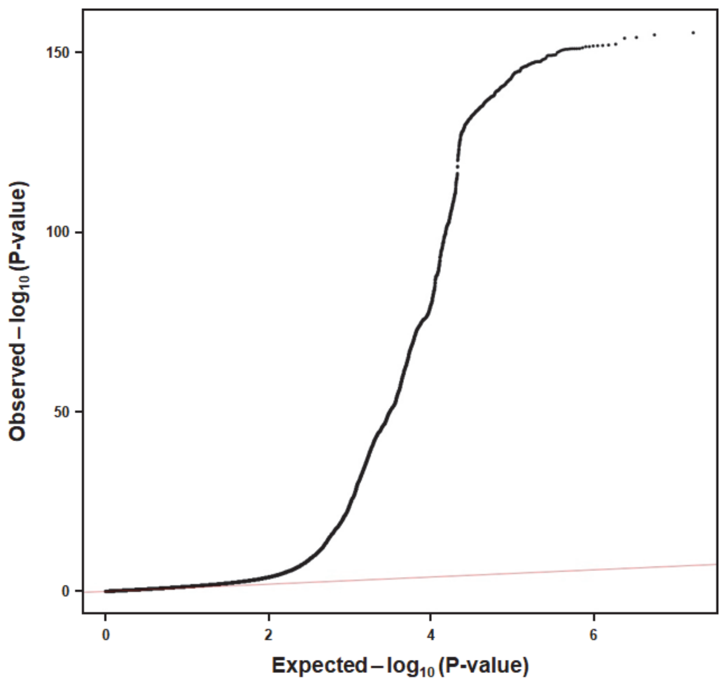

Supplementary Figure 2. Quantile-quantile plots for results of GWAS meta-analysis with the UK Biobank and TAGC. The observed versus the expected P-values from the Z-score meta-analysis are shown. The genomic control factor (λ) in the meta-analysis was 1.33 and the LD Score intercept was 1.069 (SE=0.015), suggesting that any inflation of test statistics was more likely due to many small genetic effects rather than population structure. The meta-analysis for asthma included a total of 88,486 cases and 447,859 controls from the UK Biobank (64,538 asthma cases and 329,321 controls) and TAGC (23,948 asthma cases and 118,538 controls) and 8,365,359 SNPs common to both datasets.

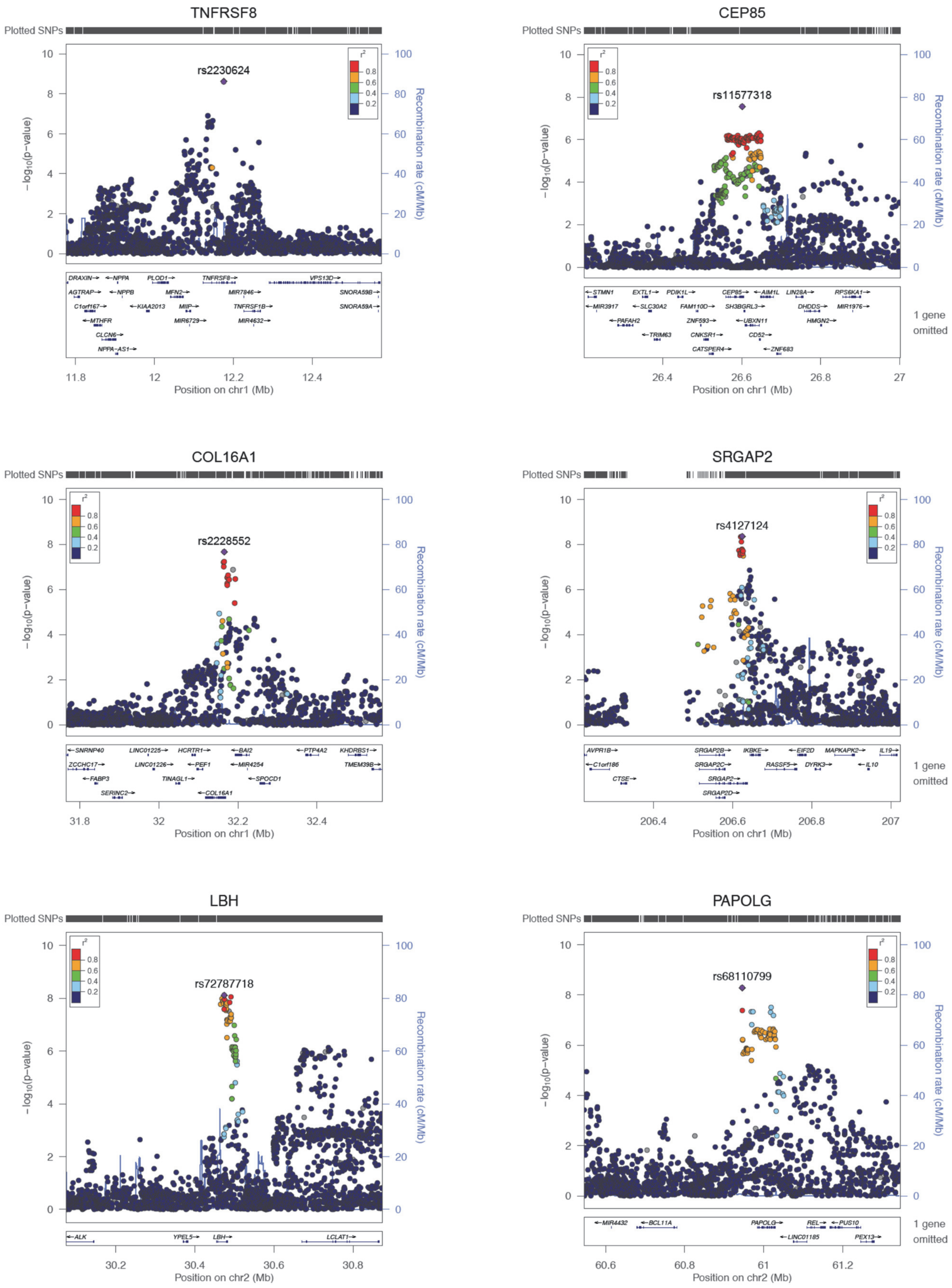
TNFRSF8
Plotted SNPs
rs2230624
−log10(p−value)
Recombination rate (cM/Mb)
Position on chr1 (Mb)
CEP85
rs11577318
1 gene omitted
Position on chr1 (Mb)
COL16A1
rs2228552
Position on chr1 (Mb)
SRGAP2
rs4127124
1 gene omitted
Position on chr1 (Mb)
LBH
rs72787718
Position on chr2 (Mb)
PAPOLG
rs68110799
1 gene omitted
Position on chr2 (Mb)

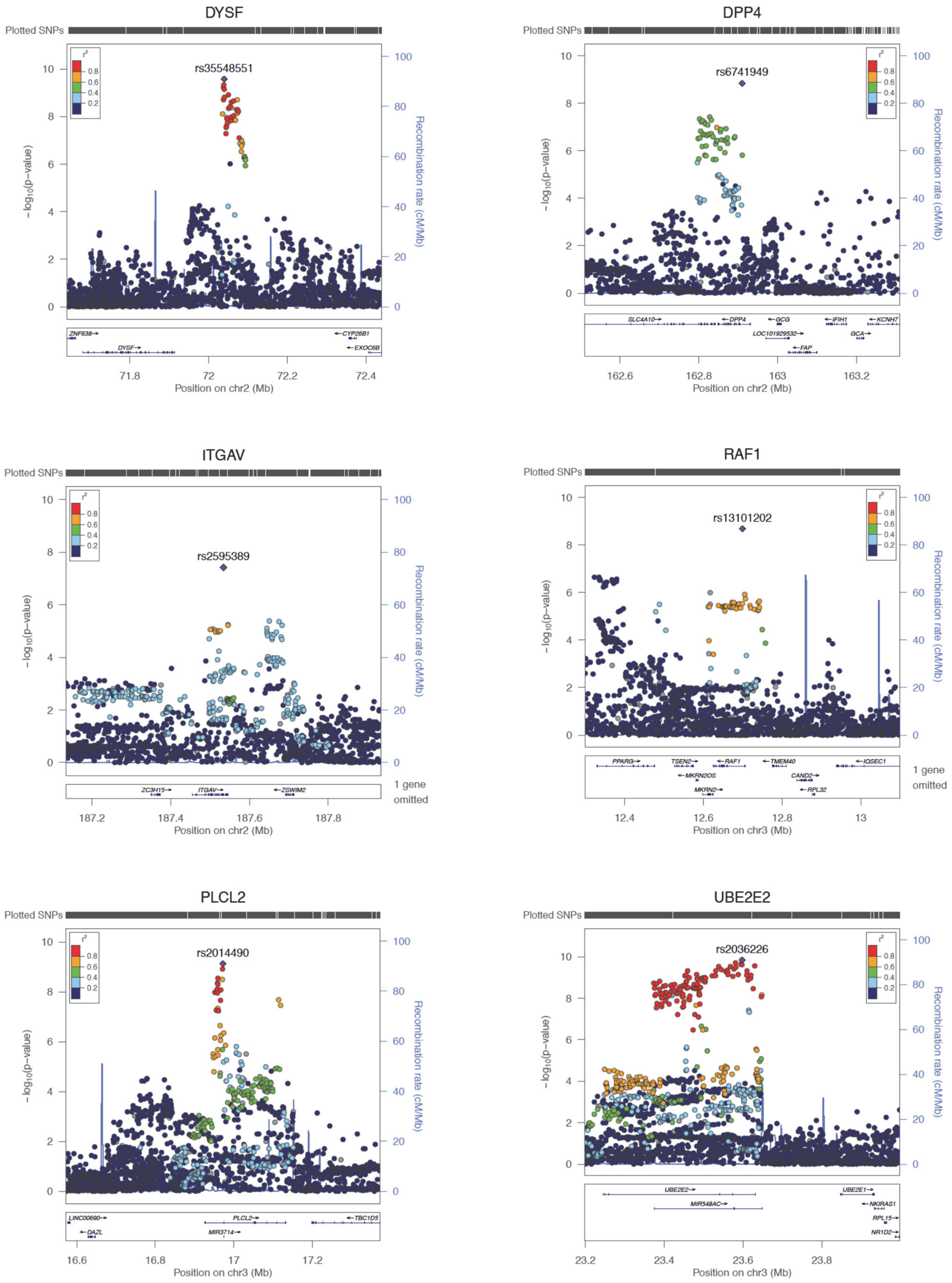
DYSF
Plotted SNPs
rs35548551
−log10(p−value)
Recombination rate (cM/Mb)
ZNF638→
DYSF→
←CYP26B1
←EXOC6B
Position on chr2 (Mb)
DPP4
rs6741949
SLC4A10→
←DPP4
←GCG
←IFIH1
←KCNH7
LOC101929532→
GCA→
←FAP
ITGAV
rs2595389
ZC3H15→
ITGAV→
←ZSWIM2
1 gene omitted
RAF1
rs13101202
PPARG→
TSEN2→
←RAF1
←TMEM40
←IQSEC1
←MKRN2OS
CAND2→
MKRN2→
←RPL32
Position on chr3 (Mb)
PLCL2
rs2014490
LINC00690→
PLCL2→
←TBC1D5
←DAZL
MIR3714→
UBE2E2
rs2036226
UBE2E2→
UBE2E1→
MIR548AC→
←NKIRAS1
RPL15→
NR1D2→

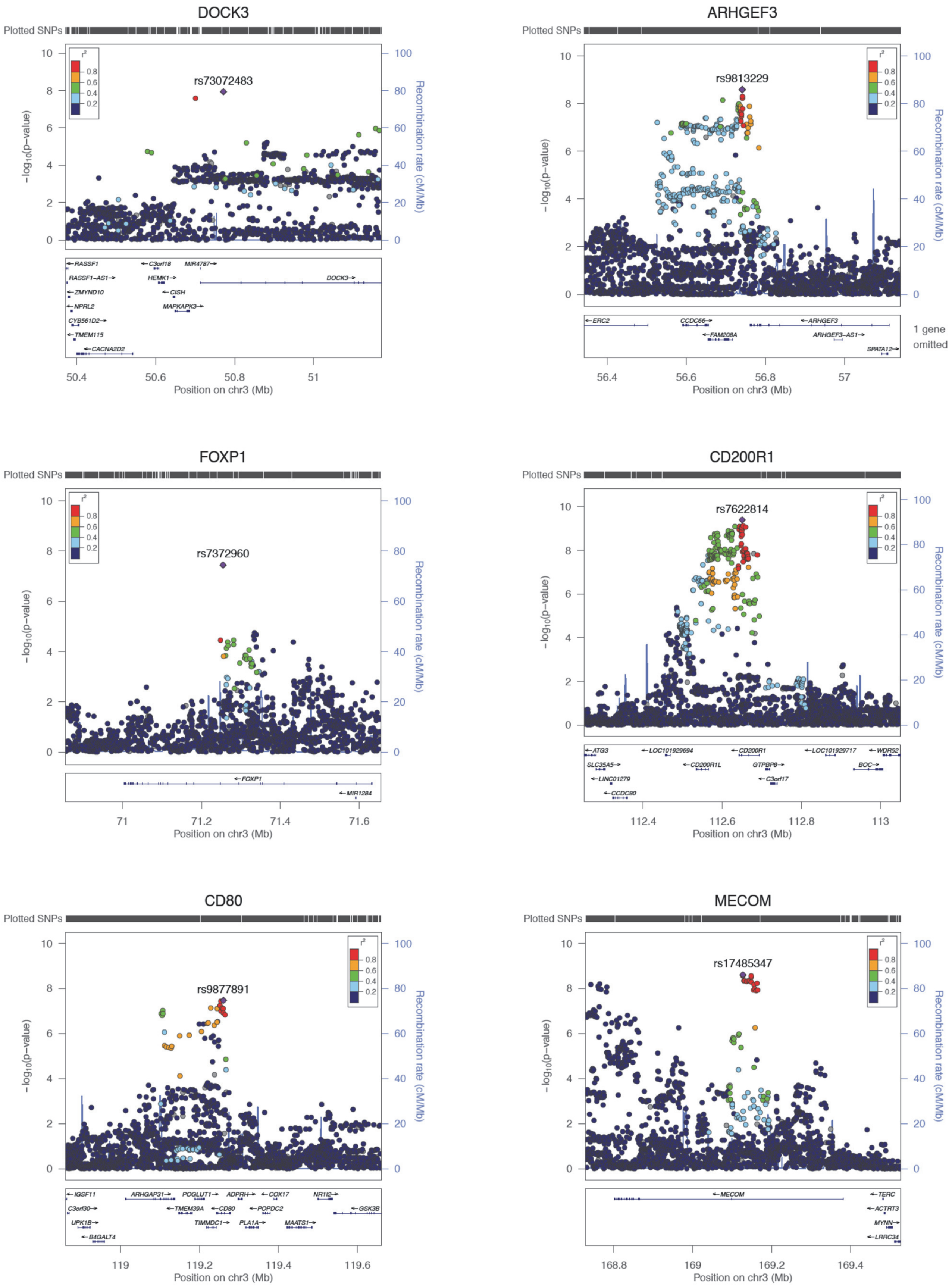

DOCK3
rs73072483
ARHGEF3
rs9813229
1 gene omitted
FOXP1
rs7372960
CD200R1
rs7622814
CD80
rs9877891
MECOM
rs17485347
Plotted SNPs
−log10(p−value)
Recombination rate (cM/Mb)
r²
Position on chr3 (Mb)
RASSF1
RASSF1−AS1
ZMYND10
NPRL2
CYB561D2
TMEM115
CACNA2D2
C3orf18
HEMK1
CISH
MAPKAPK3
MIR4787
DOCK3
ERC2
CCDC66
FAM208A
ARHGEF3
ARHGEF3−AS1
SPATA12
FOXP1
MIR1284
ATG3
SLC35A5
LINC01279
CCDC80
LOC101929694
CD200R1L
CD200R1
GTPBP8
C3orf17
LOC101929717
WDR52
BOC
IGSF11
C3orf30
UPK1B
B4GALT4
ARHGAP31
TMEM39A
POGLUT1
TIMMDC1
CD80
ADPRH
PLA1A
POPDC2
COX17
MAATS1
NR1I2
GSK3B
MECOM
TERC
ACTRT3
MYNN
LRRC34

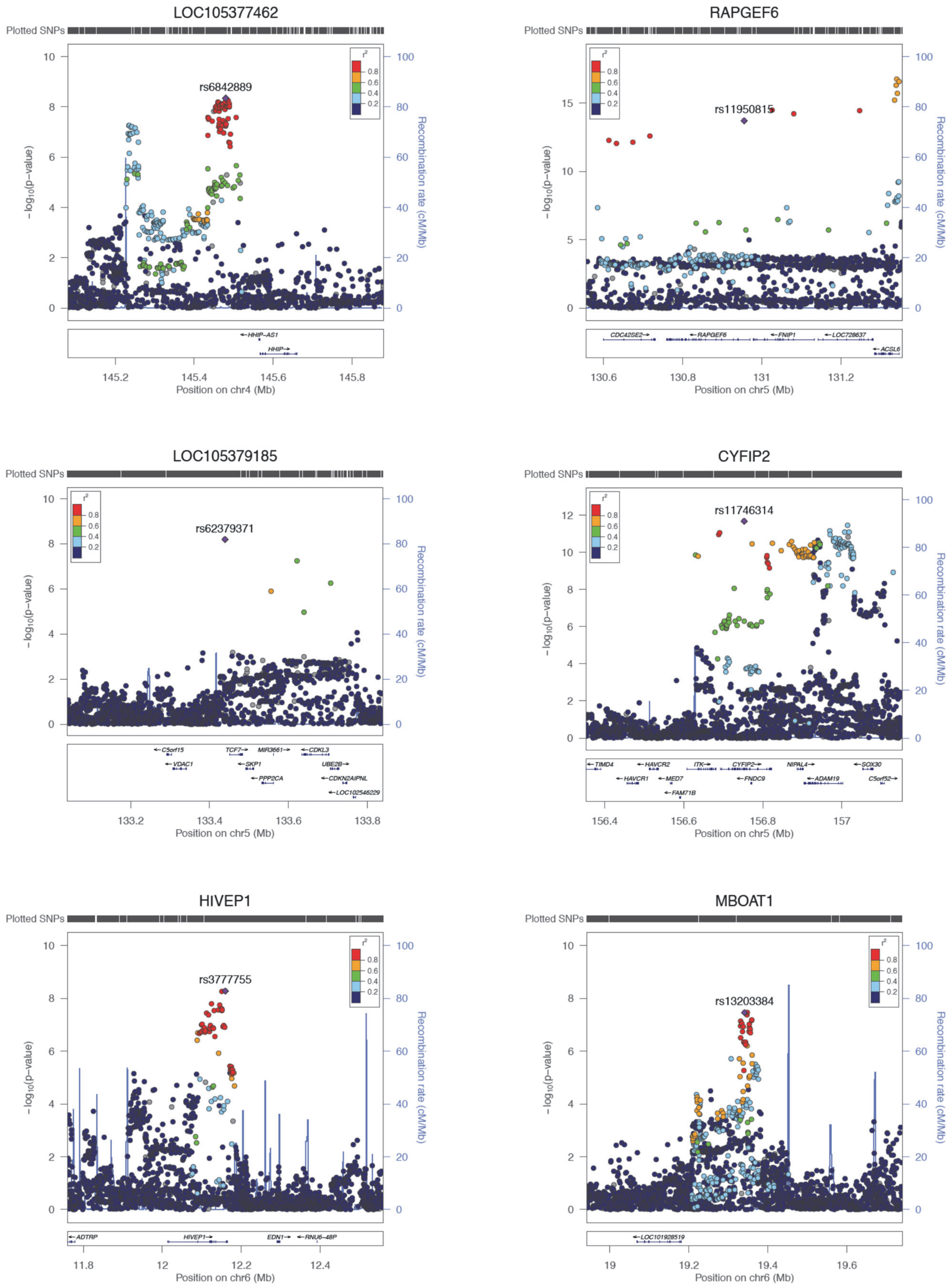

LOC105377462
rs6842889
HHIP-AS1
HHIP
Position on chr4 (Mb)
RAPGEF6
rs11950815
CDC42SE2
RAPGEF6
FNIP1
LOC728637
ACSL6
Position on chr5 (Mb)
LOC105379185
rs62379371
C5orf15
VDAC1
TCF7
SKP1
PPP2CA
MIR3661
CDKL3
UBE2B
CDKN2AIPNL
LOC102546229
Position on chr5 (Mb)
CYFIP2
rs11746314
TIMD4
HAVCR1
HAVCR2
MED7
FAM71B
ITK
CYFIP2
FNDC9
NIPAL4
ADAM19
SOX30
C5orf52
Position on chr5 (Mb)
HIVEP1
rs3777755
ADTRP
HIVEP1
EDN1
RNU6-48P
Position on chr6 (Mb)
MBOAT1
rs13203384
LOC101928519
Position on chr6 (Mb)
Plotted SNPs
−log10(p−value)
Recombination rate (cM/Mb)

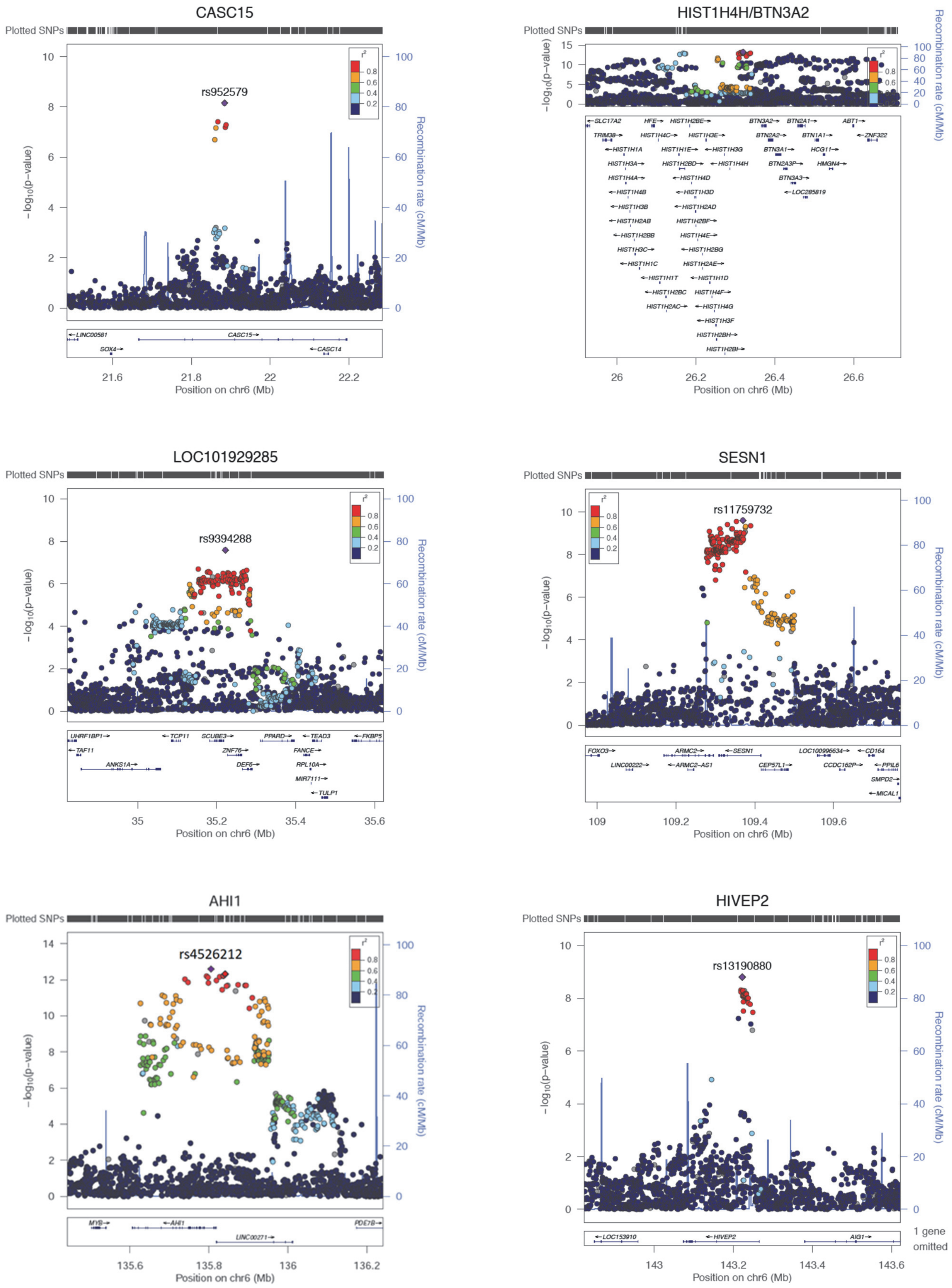
CASC15
Plotted SNPs
rs952579
−log10(p−value)
Recombination rate (cM/Mb)
LINC00581
CASC15
SOX4
CASC14
Position on chr6 (Mb)
HIST1H4H/BTN3A2
Plotted SNPs
−log10(p−value)
Recombination rate (cM/Mb)
Position on chr6 (Mb)
LOC101929285
Plotted SNPs
rs9394288
−log10(p−value)
Recombination rate (cM/Mb)
Position on chr6 (Mb)
SESN1
Plotted SNPs
rs11759732
−log10(p−value)
Recombination rate (cM/Mb)
Position on chr6 (Mb)
AHI1
Plotted SNPs
rs4526212
−log10(p−value)
Recombination rate (cM/Mb)
MYB
AHI1
LINC00271
PDE7B
Position on chr6 (Mb)
HIVEP2
Plotted SNPs
rs13190880
−log10(p−value)
Recombination rate (cM/Mb)
LOC153910
HIVEP2
AIG1
1 gene omitted
Position on chr6 (Mb)

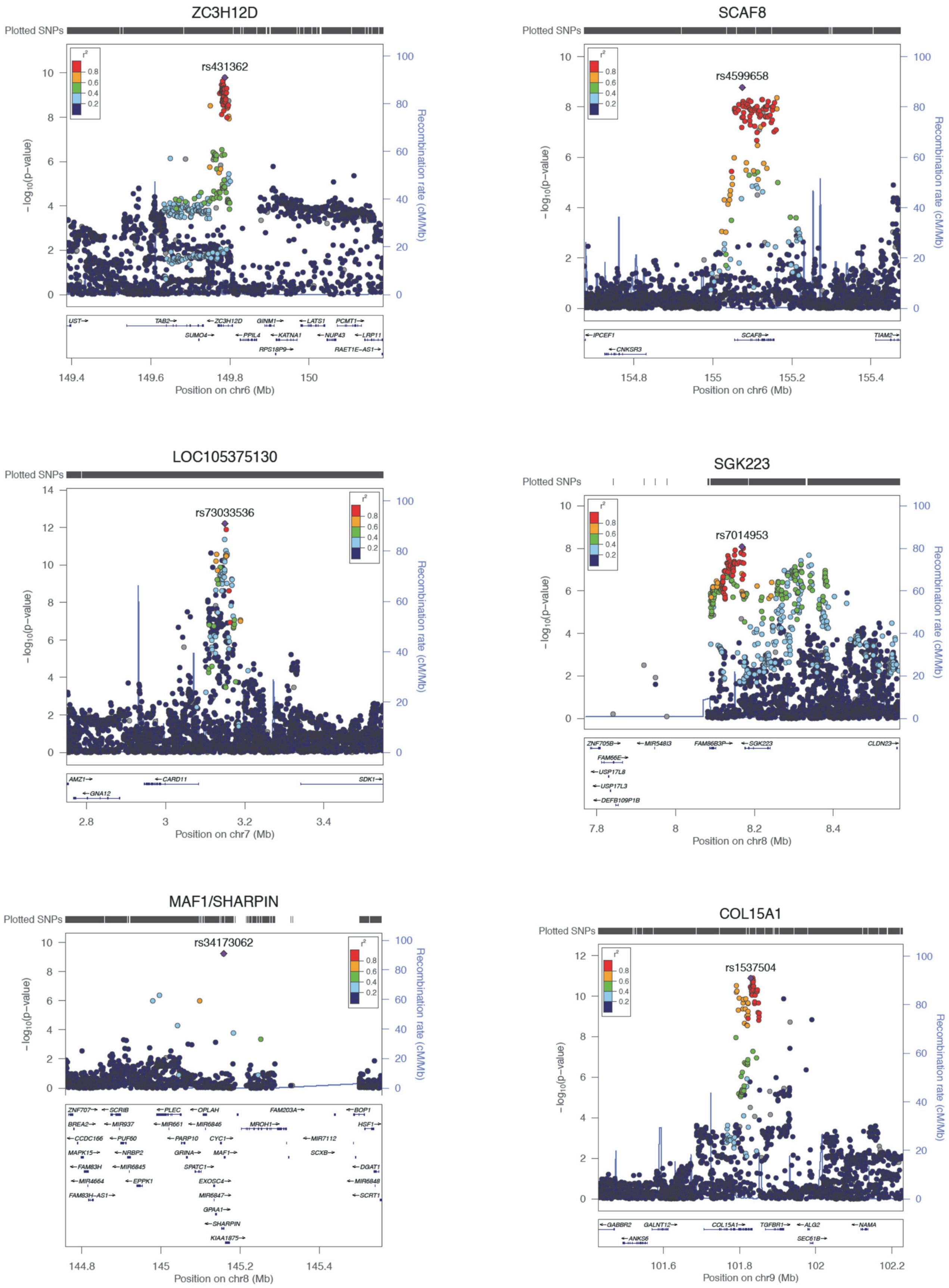
ZC3H12D
rs431362
Position on chr6 (Mb)
SCAF8
rs4599658
Position on chr6 (Mb)
LOC105375130
rs73033536
Position on chr7 (Mb)
SGK223
rs7014953
Position on chr8 (Mb)
MAF1/SHARPIN
rs34173062
Position on chr8 (Mb)
COL15A1
rs1537504
Position on chr9 (Mb)
Plotted SNPs
−log10(p−value)
Recombination rate (cM/Mb)

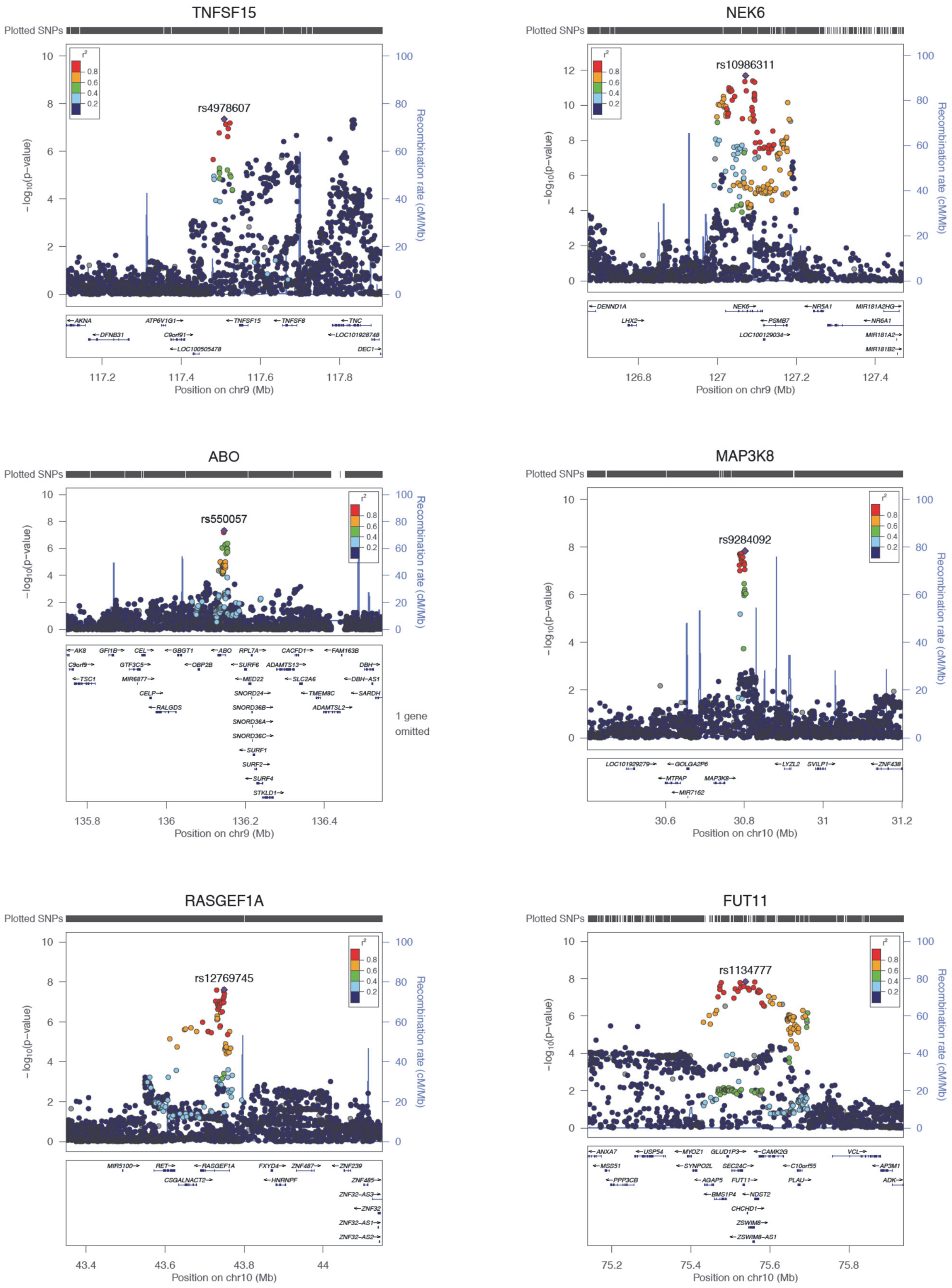
TNFSF15
rs4978607
Position on chr9 (Mb)
NEK6
rs10986311
Position on chr9 (Mb)
ABO
rs550057
1 gene omitted
Position on chr9 (Mb)
MAP3K8
rs9284092
Position on chr10 (Mb)
RASGEF1A
rs12769745
Position on chr10 (Mb)
FUT11
rs1134777
Position on chr10 (Mb)
Plotted SNPs
Recombination rate (cM/Mb)

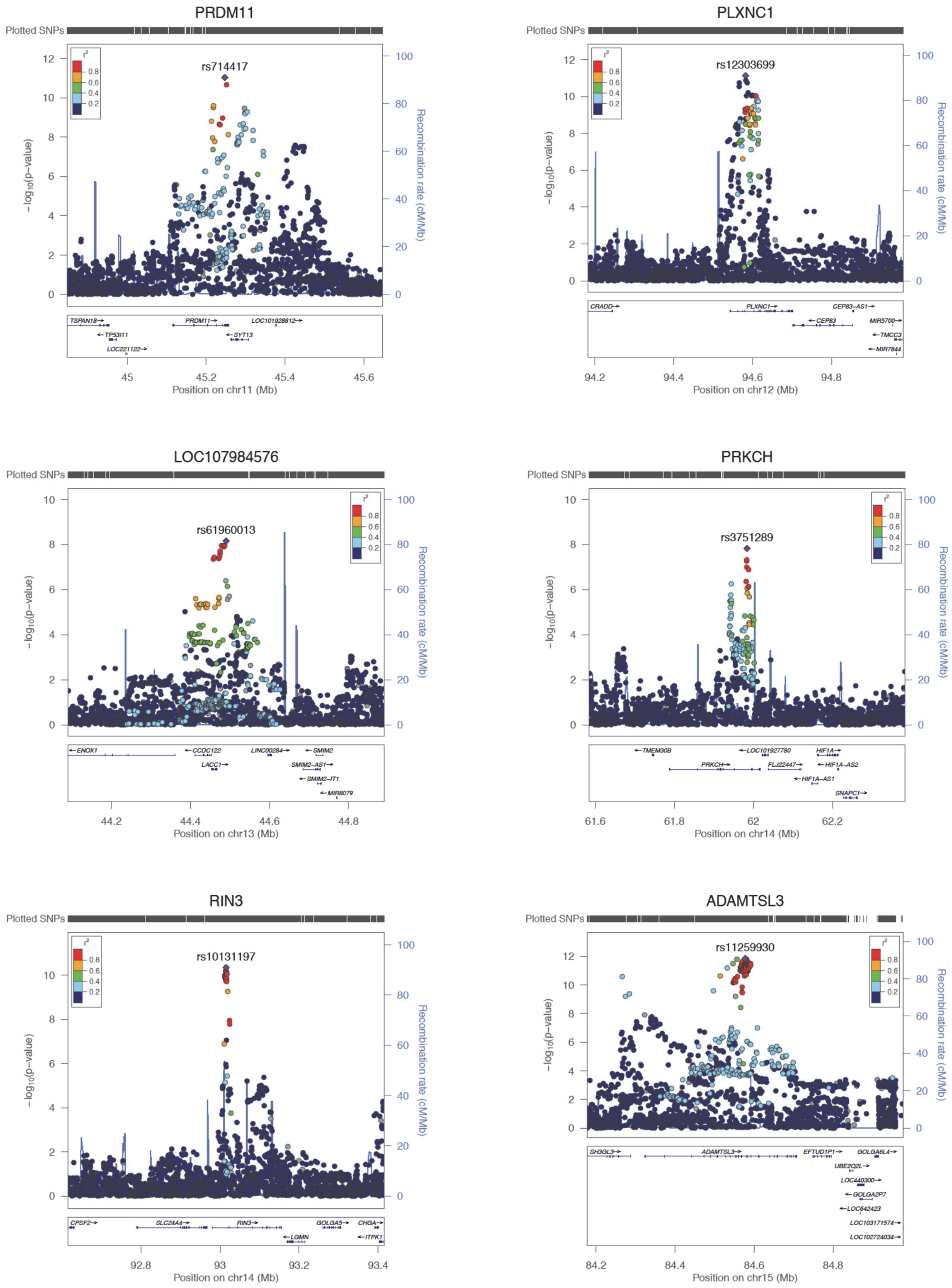

PRDM11
rs714417
Position on chr11 (Mb)
PLXNC1
rs12303699
Position on chr12 (Mb)
LOC107984576
rs61960013
Position on chr13 (Mb)
PRKCH
rs3751289
Position on chr14 (Mb)
RIN3
rs10131197
Position on chr14 (Mb)
ADAMTSL3
rs11259930
Position on chr15 (Mb)
Plotted SNPs
$-\log_{10}$(p-value)
Recombination rate (cM/Mb)
r^2
TSPAN18
TP53I11
LOC221122
PRDM11
SYT13
LOC101928812
CRADD
PLXNC1
CEP83-AS1
CEP83
MIR5700
TMCC3
MIR7844
ENOX1
CCDC122
LACC1
LINC00284
SMIM2
SMIM2-AS1
SMIM2-IT1
MIR8079
TMEM30B
PRKCH
LOC101927780
FLJ22447
HIF1A
HIF1A-AS2
HIF1A-AS1
SNAPC1
CPSF2
SLC24A4
RIN3
LGMN
GOLGA5
CHGA
ITPK1
SH3GL3
ADAMTSL3
EFTUD1P1
GOLGA6L4
UBE2Q2L
LOC440300
GOLGA2P7
LOC642423
LOC103171574
LOC102724034

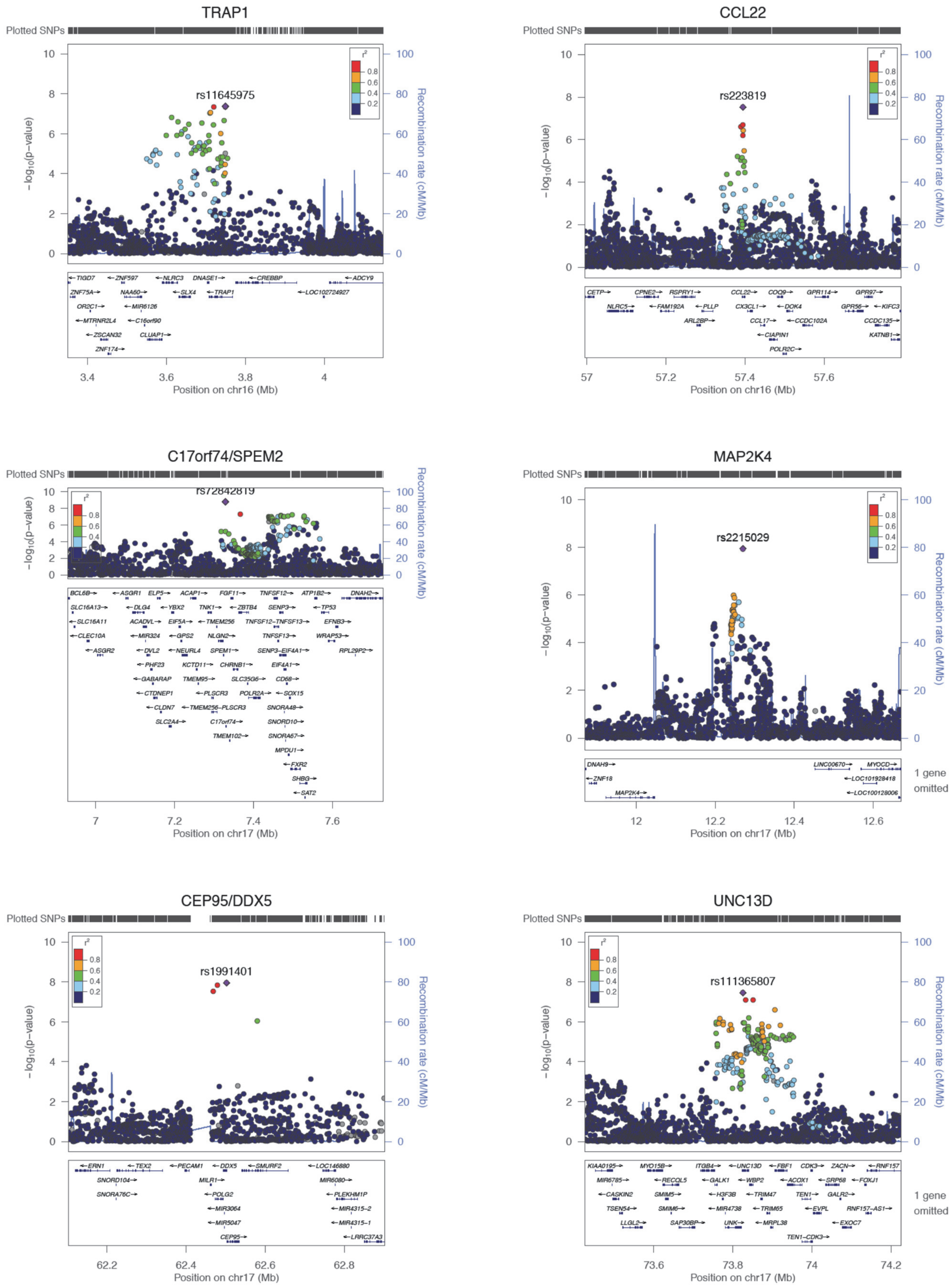
TRAP1
Plotted SNPs
rs11645975
−log10(p−value)
Recombination rate (cM/Mb)
Position on chr16 (Mb)
CCL22
Plotted SNPs
rs223819
−log10(p−value)
Recombination rate (cM/Mb)
Position on chr16 (Mb)
C17orf74/SPEM2
Plotted SNPs
rs72842819
−log10(p−value)
Recombination rate (cM/Mb)
Position on chr17 (Mb)
MAP2K4
Plotted SNPs
rs2215029
−log10(p−value)
Recombination rate (cM/Mb)
1 gene omitted
Position on chr17 (Mb)
CEP95/DDX5
Plotted SNPs
rs1991401
−log10(p−value)
Recombination rate (cM/Mb)
Position on chr17 (Mb)
UNC13D
Plotted SNPs
rs111365807
−log10(p−value)
Recombination rate (cM/Mb)
1 gene omitted
Position on chr17 (Mb)

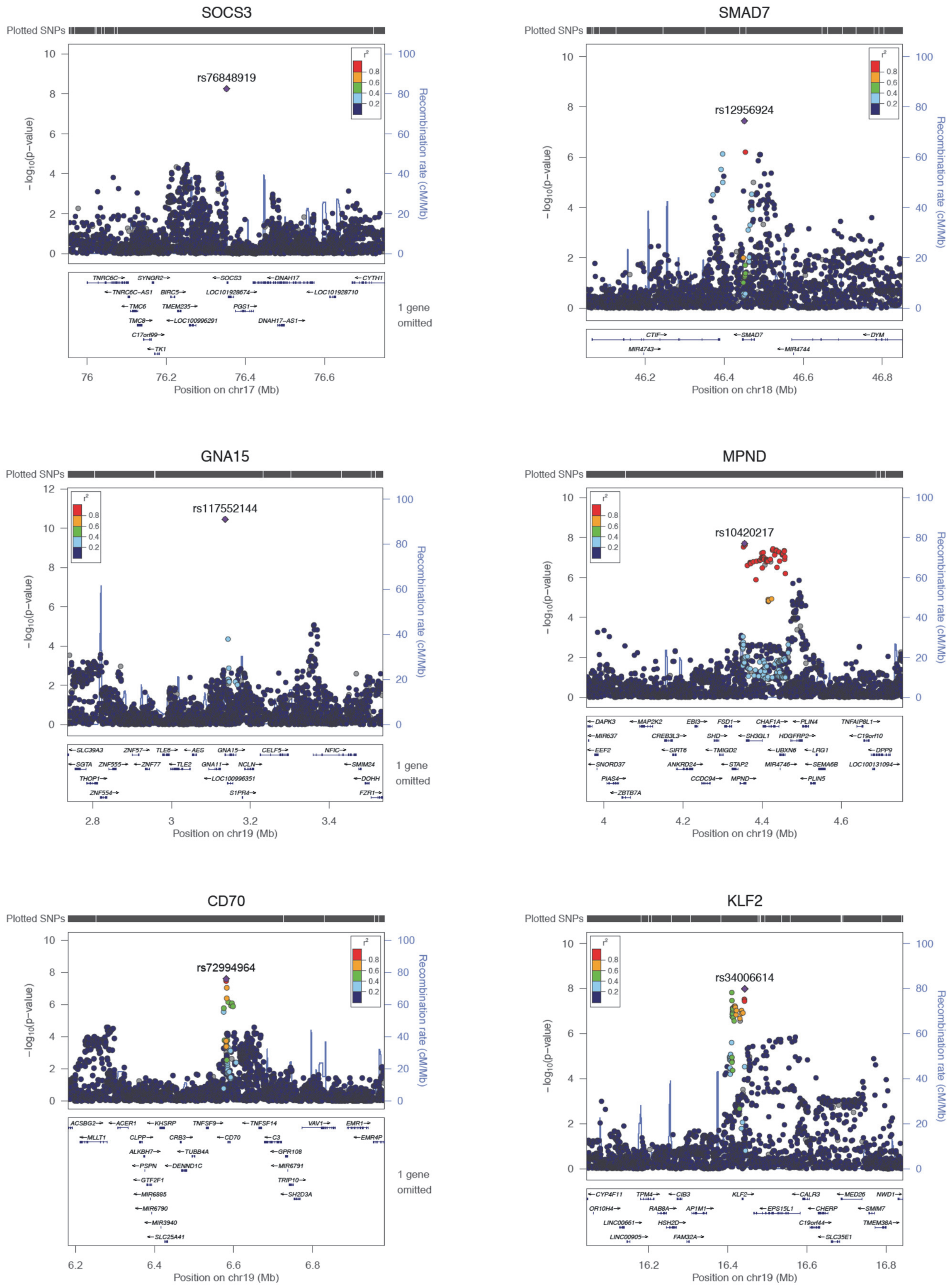
SOCS3
Plotted SNPs
rs76848919
−log10(p−value)
Recombination rate (cM/Mb)
r²
TNRC6C→
SYNGR2→
←SOCS3
←DNAH17
←CYTH1
←TNRC6C−AS1
BIRC5→
LOC101928674→
←LOC101928710
←TMC6
TMEM235→
PGS1→
TMC8→
←LOC100996291
DNAH17−AS1→
C17orf99→
←TK1
1 gene omitted
Position on chr17 (Mb)
SMAD7
Plotted SNPs
rs12956924
−log10(p−value)
Recombination rate (cM/Mb)
r²
CTIF→
←SMAD7
←DYM
MIR4743→
←MIR4744
Position on chr18 (Mb)
GNA15
Plotted SNPs
rs117552144
−log10(p−value)
Recombination rate (cM/Mb)
r²
←SLC39A3
ZNF57→
TLE6→
←AES
GNA15→
CELF5→
NFIC→
←SGTA
ZNF555→
←ZNF77
←TLE2
GNA11→
NCLN→
←SMIM24
THOP1→
←LOC100996351
←DOHH
ZNF554→
S1PR4→
FZR1→
1 gene omitted
Position on chr19 (Mb)
MPND
Plotted SNPs
rs10420217
−log10(p−value)
Recombination rate (cM/Mb)
r²
←DAPK3
←MAP2K2
EBI3→
FSD1→
CHAF1A→
←PLIN4
TNFAIP8L1→
←MIR637
CREB3L3→
SHD→
←SH3GL1
HDGFRP2→
←C19orf10
←EEF2
←SIRT6
←TMIGD2
←UBXN6
←LRG1
←DPP9
←SNORD37
ANKRD24→
←STAP2
MIR4746→
←SEMA6B
LOC100131094→
PIAS4→
CCDC94→
MPND→
←PLIN5
←ZBTB7A
Position on chr19 (Mb)
CD70
Plotted SNPs
rs72994964
−log10(p−value)
Recombination rate (cM/Mb)
r²
ACSBG2→
←ACER1
←KHSRP
TNFSF9→
←TNFSF14
VAV1→
EMR1→
←MLLT1
CLPP→
CRB3→
←CD70
←C3
←EMR4P
ALKBH7→
←TUBB4A
←GPR108
←PSPN
←DENND1C
←MIR6791
←GTF2F1
TRIP10→
←MIR6885
←SH2D3A
←MIR6790
←MIR3940
←SLC25A41
1 gene omitted
Position on chr19 (Mb)
KLF2
Plotted SNPs
rs34006614
−log10(p−value)
Recombination rate (cM/Mb)
r²
←CYP4F11
TPM4→
←CIB3
KLF2→
←CALR3
←MED26
NWD1→
OR10H4→
RAB8A→
AP1M1→
←EPS15L1
←CHERP
←SMIM7
LINC00661→
HSH2D→
C19orf44→
TMEM38A→
LINC00905→
FAM32A→
←SLC35E1
Position on chr19 (Mb)

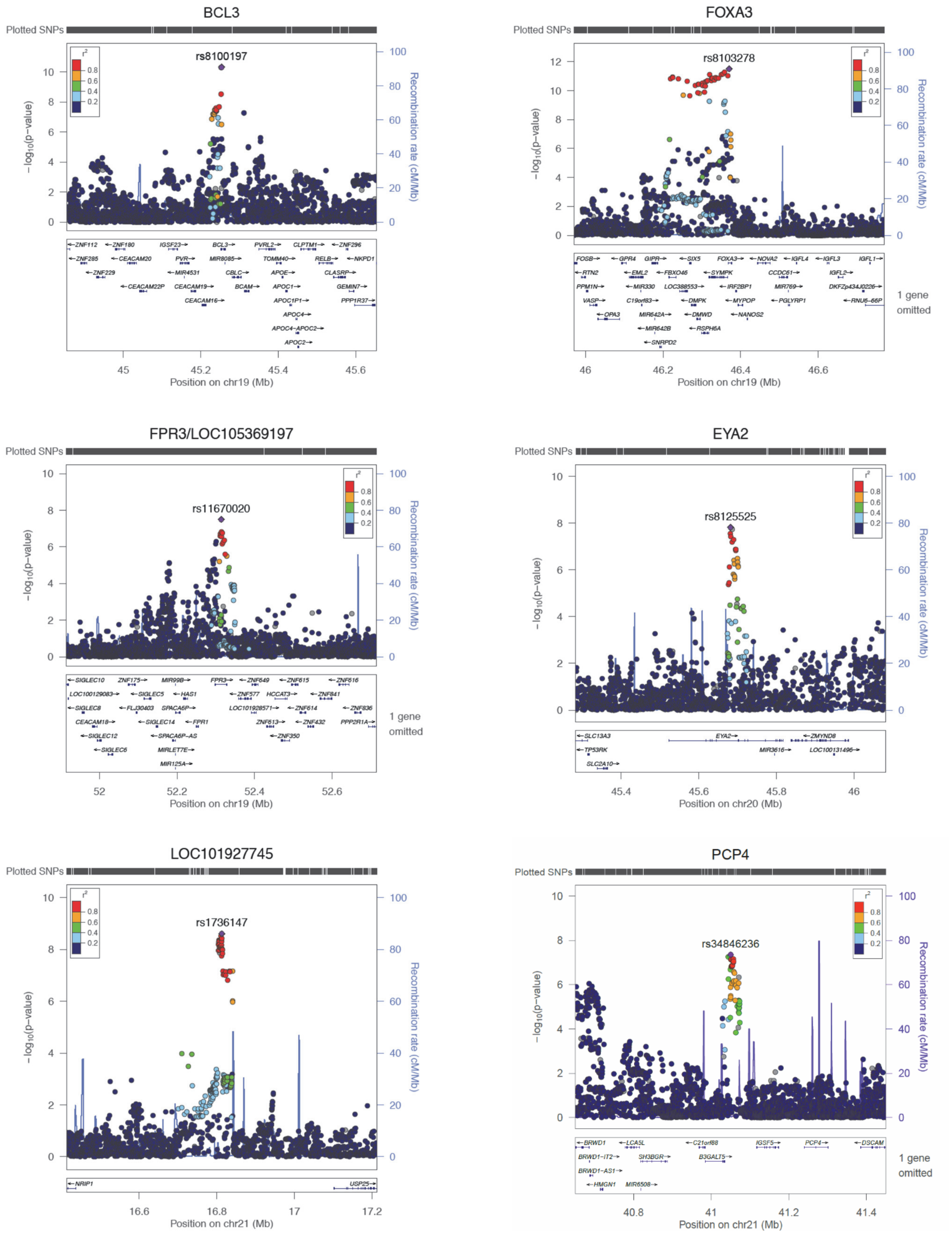

Supplementary Figure 3. Regional plots of 66 previously unknown loci identified for asthma in GWAS and meta-analyses with the UK Biobank and TAGC. 58 loci were identified as being significantly

associated with asthma in the meta-analysis of the UK Biobank and TAGC with the gene(s) nearest to the lead SNP indicated above the regional plot. 8 loci (*RAF1*, *LOC105379185*, *LOC107984576*, *CEP95-DDX5*, *SOCS3*, *SMAD7*, *GNA15*, *MPND*) were genome-wide significant in the GWAS for asthma in the UK Biobank alone. Each region is centered on the lead SNP (purple diamond) and the genes in the interval are indicated in the bottom panel. The degree of linkage disequilibrium (LD) between the lead SNP and other variants is shown as r^2 values according to the color-coded legend in the box.

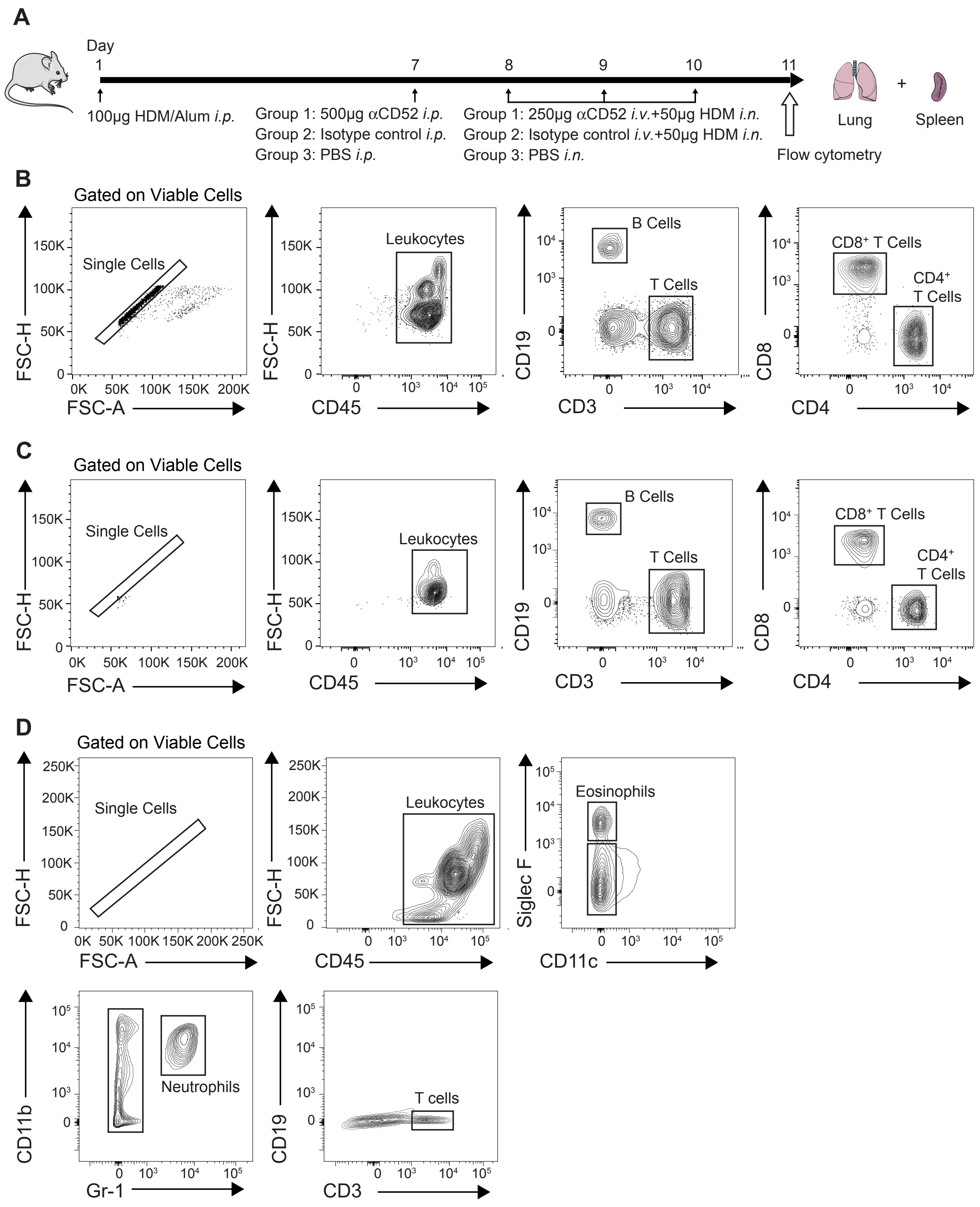

Supplemental Figure 4. Gating strategy used to quantitate immune cells by flow cytometry. (A) Female BALB/cByJ mice were immunized on day 1 with 100µg of house dust mite (HDM) in 2mg of aluminum hydroxide (alum) by intraperitoneal (i.p.) injection. On day 7, mice were intraperitoneally administered with 500µg of either the αCD52 antibody (Group 1, red bars; n=8) or isotype control antibody (Group 2, black bars; n=9), or phosphate-buffered saline (PBS) (Group 3, white bars; n=4). On days 8, 9 and 10, mice in Groups 1 and 2 were intravenously (i.v.) administered 250µg of the αCD52 antibody or isotype control antibody, respectively, and simultaneously challenged intranasally (i.n.) with 50µg HDM. Mice in Group 3 were only challenged with PBS i.n. on days 8, 9 and 10. On day 11, mice were euthanized and relative and absolute numbers of lymphocytes in lung and spleen were quantified by means of flow cytometry (see Methods for details). Representative flow plots showing that bone marrow-derived lymphocytes in lung (B) and spleen (C) were identified by gating on CD45, followed by gating on CD19 and CD3 for B and T cells. T cells were further gated on CD4 and CD8 to quantitate these subpopulations. A similar strategy was used in bronchial alveolar lavage (D) to quantitate bone marrow-derived lymphocytes by gating on CD45, followed by gating on Siglec-F and CD11c for eosinophils, Gr-1 and CD11b for neutrophils, and CD19 and CD3 for T cells.

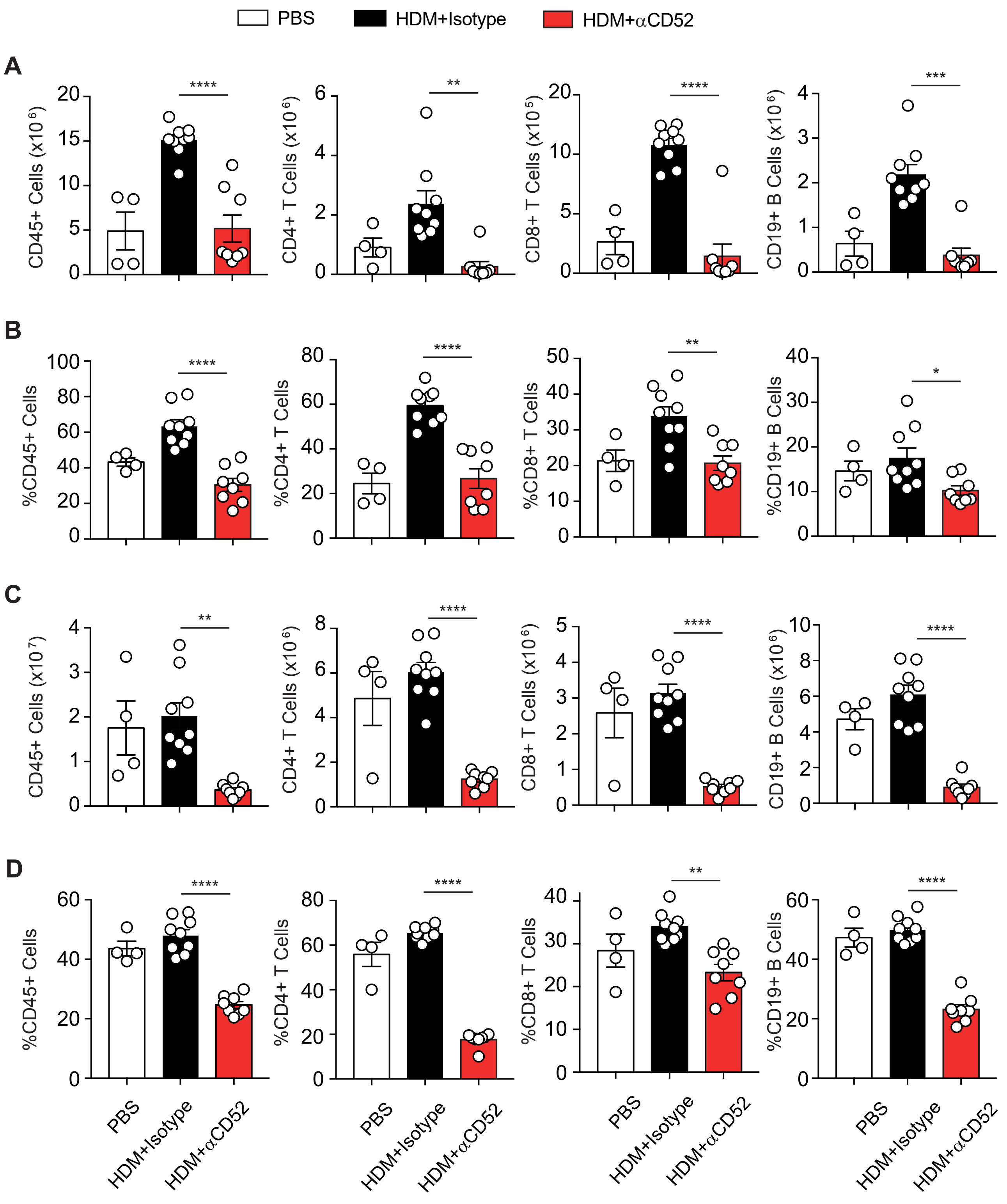

Supplemental Figure 5. Depletion of pulmonary and splenic lymphocytes by a mouse anti-CD52 (αCD52) antibody. Compared to HDM-exposed mice receiving the control isotype antibody (black bars; n=9), the number (A) and percentage (B) of CD45+ cells, CD4+ and CD8+ T cells, and CD19+ B cells were significantly lower in lungs of HDM-exposed mice administered the αCD52 antibody (red bars; n=8). The number (C) and percentage (D) of CD45+ lymphocytes, T cells, and B cells were similarly depleted in spleens of HDM-exposed mice receiving the αCD52 antibody compared to the control isotype antibody group. There were no effects in mice exposed to phosphate-buffered saline (PBS) (white bars; n=4). Data are shown as mean ± SE. *$P<0.05$, **$P<0.005$; ***$P<0.0005$; ****$P<0.0001$.
