# Supplementary material for: Genome-wide analysis highlights contribution of immune system pathways to the genetic architecture of asthma
Source: Nat Commun. 2020 Apr 15;11:1776. doi: 10.1038/s41467-020-15649-3 (PMC7160128; doi:10.1038/s41467-020-15649-3)
Supplement: Supplementary file 2 — Description of Additional Supplementary Files [file 41467_2020_15649_MOESM2_ESM.pdf]

Title: Supplementary Data 1:
Description: Description of individual cohorts in the TAGC.

Title: Supplementary Data 2:
Description: Results for 145 loci significantly associated with asthma in the GWAS analysis of the UK Biobank.

Title: Supplementary Data 3:
Description: Results for 167 loci significantly associated with asthma in GWAS meta-analysis of the UK Biobank and TAGC.

Title: Supplementary Data 4:
Description: Comparison of association results for 146 known asthma loci from previous studies and our GWAS meta-analysis with the UK Biobank and TAGC.

Title: Supplementary Data 5:
Description: Association results for 66 previously unknown loci and 3 additional loci when only subjects of European ancestry from TAGC were included in the meta-analysis.

Title: Supplementary Data 6:
Description: Combined and sex-stratified results from the UK Biobank for 212 previously known and previously unreported loci significantly associated with asthma.

Title: Supplementary Data 7:
Description: Sensitivity analyses with 66 previously unknown loci in the UK Biobank.

Title: Supplementary Data 8:
Description: Association results of 66 previously unknown loci with lung function traits in the UK Biobank.

Title: Supplementary Data 9:
Description: PheWAS analysis for previously unknown asthma loci using Phenoscanner.

Title: Supplementary Data 10:
Description: Results of GARFIELD regulatory region enrichment analyses with SNPs associated with asthma at various significance thresholds in GWAS meta-analysis of the UK Biobank and TAGC (peaks).

Title: Supplementary Data 11:
Description: Results of DEPICT gene set enrichment analysis with SNPs significantly associated with asthma in GWAS meta-analysis of the UK Biobank and TAGC.

Title: Supplementary Data 12:
Description: Results of DEPICT tissue enrichment analysis with SNPs significantly associated with asthma in GWAS meta-analysis of the UK Biobank and TAGC.

Title: Supplementary Data 13:
Description: Multi-tissue cis-eQTLs at previously unknown loci for asthma.
